# Role of Oxygen Supply in Macrophages in a Model of Simulated Orthodontic Tooth Movement

**DOI:** 10.1155/2020/5802435

**Published:** 2020-07-29

**Authors:** Agnes Schröder, Leonie Barschkies, Jonathan Jantsch, Peter Proff, Lina Gölz, James Deschner, Christian Kirschneck

**Affiliations:** ^1^Department of Orthodontics, University Hospital Regensburg, 93053 Regensburg, Germany; ^2^Institute of Clinical Microbiology and Hygiene, University Hospital Regensburg, 93053 Regensburg, Germany; ^3^Department of Orthodontics, University of Erlangen-Nuremberg, 91054 Erlangen, Germany; ^4^Department of Periodontology and Operative Dentistry, University of Mainz, 55131 Mainz, Germany

## Abstract

Apart from periodontal ligament fibroblasts, immune cells like macrophages also play an important mediating role in orthodontic tooth movement (OTM). Upon orthodontic force application to malpositioned teeth, macrophages in the periodontal ligament get exposed to both mechanical strain and hypoxic conditions (via a compression of blood vessels). In this study, we assessed the relative impact of orthodontically induced mechanical strain and hypoxic conditions on macrophages for the mediation and regulation of OTM. Macrophages were stimulated with physiological orthodontic compressive forces of 2 g/cm^2^ for 4 h and 24 h on gas-impermeable or gas-permeable cell culture plates under normoxic or hypoxic cell culture conditions. We quantified expression of genes involved in inflammation (*Tnf*, *Il-6*, and *Cox-2*), extracellular remodelling (*Mmp-9*), and angiogenesis (*Vegf*) by RT-qPCR. Furthermore, we analysed HIF-1*α*, prostaglandin-E2, and VEGF protein expression via immunoblotting or ELISA. Mechanical strain and oxygen supply both differentially affected expression of genes and proteins involved in inflammation and angiogenesis. In this context, we found that HIF-1*α* protein levels were elevated by combined mechanical strain and hypoxic conditions, whereas gas-permeable plates providing sufficient oxygen supply prevented HIF-1*α* stabilization at the protein level after pressure application on macrophages. Our results thus indicate that macrophages involved in the mediation of OTM are affected by and respond differently to hypoxic conditions and mechanical compressive strain, which occur concomitantly during OTM, than periodontal ligament fibroblasts (PDLF), thus indicating different roles of these cells in the regulation of OTM at the cellular-molecular level. We further observed that contrary to PDLF HIF-1*α* stabilization in macrophages is rather induced via the decreased oxygen supply associated with OTM than via mechanotransduction by mechanical strain.

## 1. Introduction

Carl Sandstedt examined tissue remodelling during orthodontic treatments aimed at correcting malocclusions and malpositioned teeth over 100 years ago and found that the alveolar bone adapts to the pressure and tension zones created within the periodontal ligament by the application of therapeutic orthodontic forces to teeth [1]. Since then, cellular responses during orthodontic force application have been researched at the molecular level. It has been reported that a compression of blood vessels within the periodontal ligament occurs during orthodontic tooth movement (OTM) leading to a decreased local perfusion and concomitant reduction in oxygen supply (hypoxia) [2]. Cytokines and other inflammatory markers are secreted into the periodontal tissue by periodontal fibroblasts [3, 4] or immune cells [5] to attract additional leukocytes and macrophages [6, 7] inducing a “pseudoinflammatory process” [1]. This process is characterized by a promotion of inflammation but may also accelerate other noninflammatory processes mediated by periodontal ligament cells [1]. Next to fibroblasts, which make up the main cell population in the periodontal ligament, immune cells like macrophages are also present, which will be exposed to mechanical strain and changing oxygen supply during orthodontic tooth movement [8]. It is now known that macrophages interact with fibroblasts of the periodontal ligament and promote tooth movement by modulating differentiation from osteoclast progenitor cells into bone resorbing osteoclasts [8]. Furthermore, macrophages secrete a variety of cytokines such as tumor necrosis factor (TNF) or interleukin 6 (IL-6) that stimulate bone resorption by increasing receptor activator of NF-*κ*B ligand (RANKL) expression [9, 10]. In order to adapt to the new environmental parameters and reduced oxygen supply, angiogenic factors are increasingly expressed by periodontal ligament cells, so that an adequate blood supply, which is associated with improved oxygen saturation, can be restored [11]. By stabilizing hypoxia inducible factor 1*α* (HIF-1*α*) protein, more than 100 different target genes with different functions can be activated [12]. HIF-1*α* binds to the HIF-responsive elements in the promoter or enhancer region of target genes and thus stimulates transcription. To counteract hypoxia, HIF-1*α* activates some genes that code for improved oxygen transport, angiogenesis, vasodilatation, and anaerobic glycolysis [13].

In this work, we focus on the HIF 1 target genes *vascular endothelial growth factor* (*Vegf*) and *cyclooxygenase-2* (*Cox-2*). VEGF and COX-2 are important mediators for angiogenesis [14] and inflammation [15] and are upregulated in periodontal ligament cells during mechanical strain as occurring during OTM [4, 16, 17]. *Cox-2* is increasingly expressed, particularly during mechanical strain and inflammation in order to catalyse the conversion of arachidonic acid to prostaglandins, most prominently prostaglandin E2 [16, 18], which among others contributes to vasodilatation [11]. Furthermore, prostaglandin E2 promotes osteoclastogenesis and is involved in extracellular matrix remodelling by regulating the expression of matrix metalloproteinases [19], which are required for the degradation of the extracellular matrix [1].

Fibroblasts of the periodontal ligament stabilized HIF-1*α* protein after compressive force treatment. This stabilization was mainly due to mechanotransductive effects, whereas hypoxia played a minor role [17]. However, effects of compressive force treatment compared to oxygen supply on macrophages are so far unknown. As these immune cells also constitute an important cell population within the periodontal ligament and are involved in the regulation and instigation of OTM at the cellular-molecular level [8], the aim of this work was therefore to clarify the relative impact of orthodontically induced mechanical strain and hypoxic conditions in the periodontal ligament on macrophages for the mediation and regulation of OTM focusing on HIF-1*α* expression and its stabilization as well as on genes and proteins involved in the inflammatory processes occurring during OTM. To address this question, we used an established *in vitro* model to simulate orthodontic force application and mechanical strain as well as hypoxic conditions [16–18].

## 2. Material and Methods

### 2.1. *In Vitro* Cell Culture Experiments

Immortalised RAW264.7 macrophages (400319, CLS Cell Lines Service) were cultured in Dulbecco's modified Eagle's medium-high glucose (DMEM, D5671, Sigma-Aldrich), enriched with 10% fetal calf serum (FCS, P30-3302, PAN Biotech), 1% L-glutamine (G7513, Sigma-Aldrich), and 1% Antibiotic/Antimycotic Solution 100x (A5955, Sigma-Aldrich). The medium was replaced every two to three days. Cultivation was performed in an incubator (BBD 6220, Thermo Fisher Scientific) at 37°C and 5% CO_2_ saturation. All cell culture experiments were carried out under sterile conditions (laminar flow unit, BDK Luft- und Reinraumtechnik).

#### 2.1.1. Experiment 1: Effects of Mechanical Strain (Pressure) and Hypoxia on Macrophages

**Figure 1:**
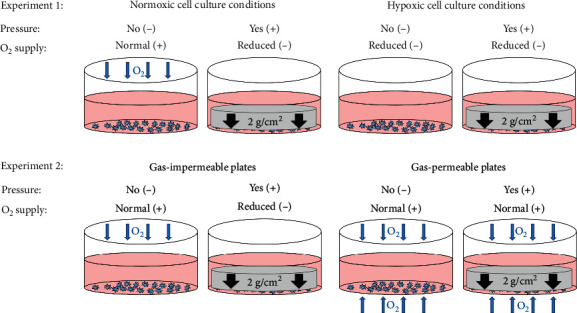
Schematic representation of different experimental settings affecting oxygen supply and mechanical strain (pressure) on adherently growing macrophages.

**Figure 2:**
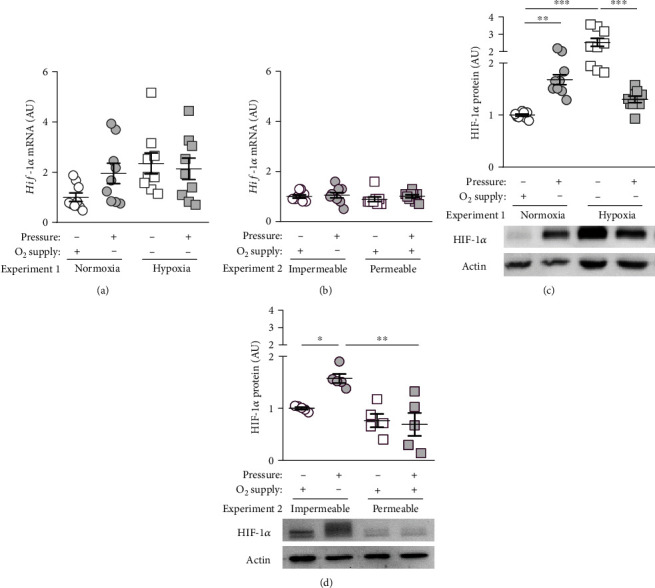
(a) *Hif-1α* gene expression under normoxic or hypoxic cell culture conditions with or without compressive force treatment. (b) *Hif-1α* gene expression on gas-impermeable or gas-permeable plates with or without pressure application. Reference genes: *Eef1a1* and *Sdha*. (c) HIF-1*α* protein expression under normoxic or hypoxic cell culture conditions with or without compressive force treatment. Below: representative immunoblot. (d) HIF-1*α* protein expression on gas-impermeable or gas-permeable plates with or without pressure application. Below: representative immunoblot. Statistics: ANOVA followed by Holm Sidak's or Tamhane's T2 multiple comparison tests; AU = arbitrary units; ^∗^*p* ≤ 0.05; ^∗∗^*p* ≤ 0.01; ^∗∗∗^*p* ≤ 0.001.

**Figure 3:**
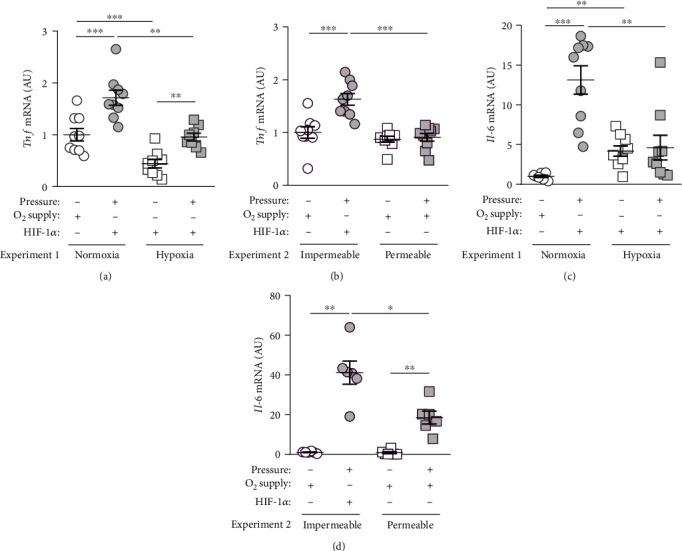
(a) *Tnf* gene expression under normoxic or hypoxic cell culture conditions with or without compressive force treatment. (b) *Tnf* gene expression on gas-impermeable or gas-permeable plates with or without pressure application. (c) *Il-6* gene expression under normoxic or hypoxic cell culture conditions with or without compressive force treatment. (d) *Il-6* gene expression on gas-impermeable or gas-permeable plates with or without pressure application. Reference genes: *Eef1a1* and *Sdha*. Statistics: ANOVA followed by Holm Sidak's or Tamhane's T2 multiple comparison tests; AU = arbitrary units; ^∗^*p* ≤ 0.05; ^∗∗^*p* ≤ 0.01; ^∗∗∗^*p* ≤ 0.001.

**Figure 4:**
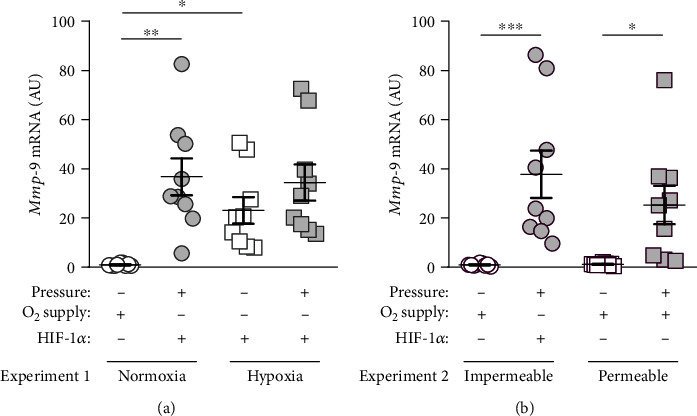
(a) *Mmp-9* gene expression under normoxic or hypoxic cell culture conditions with or without compressive force treatment. (b) *Mmp-9* gene expression on gas-impermeable or gas-permeable plates with or without pressure application. Reference genes: *Eef1a1* and *Sdha*. Statistics: ANOVA followed by Holm Sidak's or Tamhane's T2 multiple comparison tests; AU = arbitrary units; ^∗^*p* ≤ 0.05; ^∗∗^*p* ≤ 0.01; ^∗∗∗^*p* ≤ 0.001.

**Figure 5:**
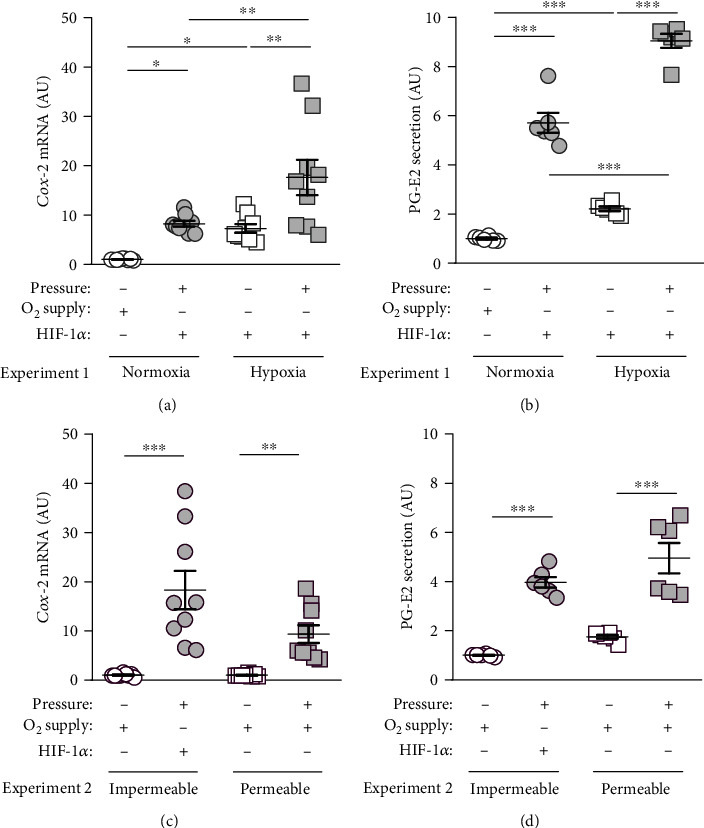
(a) *Cox-2* gene expression under normoxic or hypoxic cell culture conditions with or without compressive force treatment. (b) PG-E2 secretion under normoxic or hypoxic cell culture conditions with or without compressive force treatment. (c) *Cox-2* gene expression on gas-impermeable or gas-permeable plates with or without pressure application. (d) PG-E2 secretion on gas-impermeable or gas-permeable plates with or without pressure application. Reference genes: *Eef1a1* and *Sdha*. Statistics: ANOVA followed by Holm Sidak's or Tamhane's T2 multiple comparison tests; AU = arbitrary units; ^∗^*p* ≤ 0.05; ^∗∗^*p* ≤ 0.01; ^∗∗∗^*p* ≤ 0.001.

**Figure 6:**
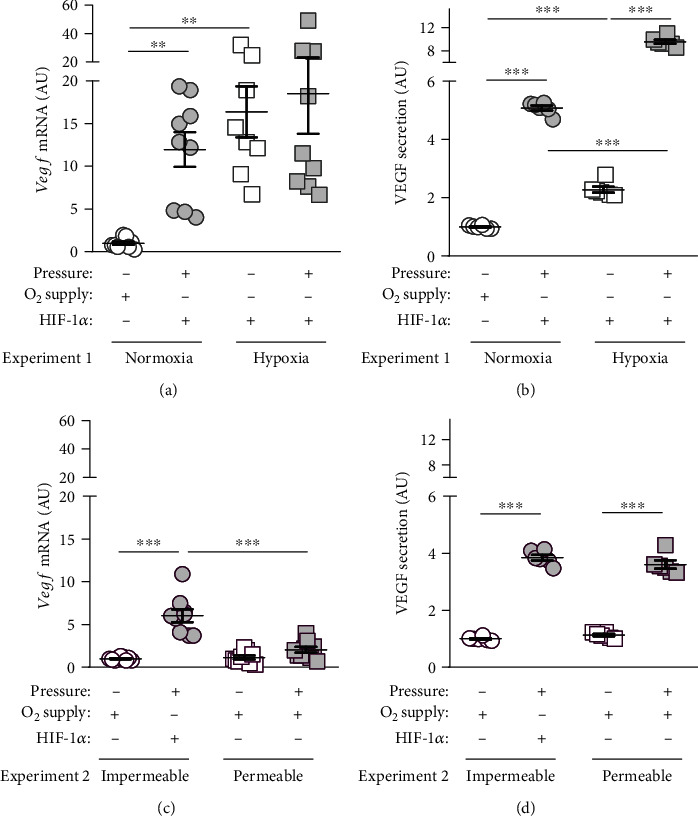
(a) *Vegf* gene expression under normoxic or hypoxic cell culture conditions with or without compressive force treatment. (b) VEGF secretion under normoxic or hypoxic cell culture conditions with or without compressive force treatment. (c) *Vegf* gene expression on gas impermeable or gas permeable plates with or without pressure application. (d) VEGF secretion on gas-impermeable or gas-permeable plates with or without pressure application. Reference genes: *Eef1a1* and *Sdha*. Statistics: ANOVA followed by Holm Sidak's or Tamhane's T2 multiple comparison tests; AU = arbitrary units; ^∗∗^*p* ≤ 0.01; ^∗∗∗^*p* ≤ 0.001.

In this experiment, we aimed to differentiate effects of mechanical strain (pressure) and hypoxia on macrophages. Therefore, approximately 250,000 RAW264.7 macrophages per ml were seeded onto sterile 6-well cell culture plates (353046, Omnilab) and cultivated for 24 h before the start of the experiment. Compressive force application was performed by using glass plates with a defined weight of 2 g/cm^2^ for additional 24 h under normoxic or hypoxic conditions according to an established and published model [7, 16, 17] (Figure 1). Hypoxic conditions were achieved by using a GasPak EZ gas development bag system with an indicator (260683, BD Biosciences) according to the manufacturer's instructions increasing CO_2_ to at least 10% within 24 h. Therefore, we also measured pH (Seven Easy in combination with Inlab Expert pro electrode, Mettler Toledo) and detected a slight, but significant, effect of CO_2_ on pH in the cell culture media (Supplemental figure 1). To further elucidate the regulatory role of HIF-1*α*, we repeated Experiment 1 with the addition of DMOG instead of creating hypoxic conditions via GasPak EZ with compressive force treatment for 4 h, as DMOG effects were only detectable after this time (Experiment 3, Supplemental information and Figures 2-7).

#### 2.1.2. Experiment 2: Effects of Mechanical Strain (Pressure) versus Hypoxic Conditions on Macrophages

To experimentally separate mechanotransductive and hypoxic effects on macrophages that occur concomitantly during OTM, we used lumox cell culture dishes (94.6077.331, Sarstedt), with oxygen-permeable membranes, so that the adherently growing cells could still be supplied with oxygen under experimental pressure application, as pressure and hypoxic conditions are induced concomitantly by the glass disc applied (Figure 1). Approximately 250,000 RAW264.7 macrophages per ml were seeded either onto conventional polystyrene plates (353046, Omnilab) or on lumox plates. After 24 h of preincubation, macrophages were either compressed using glass plates with a defined weight of 2 g/cm^2^ according to an established and published model [7, 16, 17] or left untreated for further 24 h (Figure 1). To correspond to the results from repeated Experiment 1 with the addition of DMOG, we also repeated this experiment with compressive force treatment for 4 h (Supplemental figures 8-11).

### 2.2. RNA Isolation and cDNA Synthesis

After the appropriate incubation times, cell culture medium was removed and adherent RAW264.7 macrophages were scraped off the plates in 1 ml PBS. Cell number was determined using 100 *μ*l of this suspension with a Beckman Coulter Counter Z2™ (Beckman Coulter GmbH, Krefeld, Germany) according to the manufacturer's instructions. The remaining 900 *μ*l of cell solution was centrifuged at 2,000 rpm and 4°C for 5 min. PBS supernatant was removed, and the cell pellet was dissolved in 500 *μ*l peqGold Trifast (30-2020, Peqlab) and 100 *μ*l chloroform (102445, Merck) was added. After extensive mixing for 30 s, samples were incubated for 15 min on ice and centrifuged at 13,000 rpm and 4°C for 15 min. The upper aqueous phase was transferred into 500 *μ*l isopropanol (220.842.330, VWR). After incubation at -80°C overnight, samples were centrifuged at 13,000 rpm and 4°C for 30 min. The supernatant was removed, and the pellet was washed twice with 80% ethanol (32205, Sigma-Aldrich). The pellet was resuspended in 20 *μ*l RNAse-free water (T143.5, Carl Roth), and RNA concentration was measured in a NanoPhotometer (N60, Implen). Obtained RNA was transcribed to cDNA. To this aim, a master mix was prepared consisting of 2 *μ*l 5x M-MLV buffer (M531A, Promega), 0.5 *μ*l OligodT18 primer (SO 132, Thermo Fisher Scientific), 0.5 *μ*l Random Hexamer Primer (SO142, Thermo Fisher Scientific), 0.5 *μ*l 10 mM dNTPs (L 785.2, Carl Roth), 0.5 *μ*l 40 *μ*/*μ*l RiboLock RNase Inhibitor (EO 0382, Thermo Fisher Scientific), and 0.5 *μ*l M-MLV reverse transcriptase (M1701, Promega). A standardized RNA amount of 200 ng was used for cDNA synthesis. The synthesis of cDNA was performed in a thermocycler (VWR) at 37°C for 1 h.

### 2.3. Quantitative Real-Time Polymerase Chain Reaction (RT-qPCR)

RT-qPCR amplification and quality control was performed as described before [7, 17, 20–22] with the Mastercycler® ep realplex-S Thermocycler (Eppendorf AG, Hamburg, Germany). We constructed all primers (Table 1) according to MIQE quality guidelines and criteria as described before [21]. To minimize technical errors during manual pipetting, we used a mastermix for all reactions consisting of 7.5 *μ*l SYBR Green Jumpstart Taq Readymix (S4438, Sigma-Aldrich), 5.25 *μ*l RNAse-free water (T143.5, Carl Roth), and 0.375 *μ*l forward and reverse primers (MWG eurofins, Table 1) for one reaction. 13.5 *μ*l of this mastermix was mixed with 1.5 *μ*l cDNA into each well of a 96-well TW-MT plate (712282, Biozym). Each sample was analysed in technical duplicate. Amplification was performed in 45 cycles (95°C/5 min, per cycle 95°C/10 s, 60°C/8 s, and 72°C/8 s). SYBR Green I fluorescence was quantified at 521 nm at the end of each extension step. For each primer pair and qPCR run, a no-template-control without cDNA was tested to assess a possible bias in results of primer dimers or contaminating DNA. Relative gene expression, used for statistical analysis, was calculated as 2^-*Δ*Cq^ with ΔC_q_ = C_q_ (target gene) − C_q_ (mean *Eef*1*a*1 and *Sdha*), divided by the respective arithmetic 2^-*Δ*Cq^ mean of the normoxic/lumox control group without pressure to set its relative gene expression to 1 [4, 16, 20, 23].

### 2.4. Enzyme-Linked Immunosorbent Assay (ELISA)

For the analysis of vascular endothelial growth factor (VEGF) and prostaglandin E2 (PG-E2) secretion into the cell culture supernatant, we used commercially available ELISA kits (VEGF: MBS043195, PG-E2: MBS266212, MyBioSource) according to the manufacturers' instructions.

### 2.5. Immunoblot

Proteins were isolated using CelLytic M (C2978, Sigma-Aldrich) in combination with proteinase inhibitors (87786, Thermo Fisher Scientific) on ice. Equal amounts of proteins were separated on 8% polyacrylamide gels and transferred to PVDF membranes (T830.1, Carl Roth). After blocking for 1 h in 5% milk in TBS-T, these membranes were incubated with primary HIF-1*α* antibody (10006421, Cayman, diluted 1 : 1,000) or ACTIN antibody (E1C602, EnoGene, diluted 1 : 5,000) for 1 h at room temperature. After washing three times in TBS-T, membranes were incubated with a secondary antibody (611-1302, Rockland, diluted 1 : 5,000) for 1 h. Detection was performed with luminata Forte Western HRP Substrate (WBLUF0100, Sigma-Aldrich), and pictures were taken using a Genoplex Chemiluminescence system (VWR). Densitometric analysis was done with ImageJ (ver. 1.47, Wayne Rasband, National Institutes of Health, USA).

### 2.6. Statistical Analysis

Prior to statistical analysis, all absolute data values were divided by the respective arithmetic mean of the normoxic/lumox control group without pressure to obtain normalized data values relative to these controls, set to 1. Statistical analyses were performed using GraphPad Prism 8.0. Horizontal lines in graphs represent the mean ± standard error of mean, and each symbol represents a data point. All data were tested for normal distribution and homogeneity of variance (Shapiro-Wilk test, Levene test). Datasets passing both conditions were independently compared by ordinary one-way ANOVA followed by Holm Sidak's multiple comparison tests, whereas the remaining datasets were compared by Welch-corrected ANOVAs followed by Tamhane's T2 multiple comparison tests for heterogeneous variances. All differences were considered statistically significant at *p* ≤ 0.05.

## 3. Results

### 3.1. Impact of Mechanical Strain (Pressure) and Oxygen Supply on HIF-1*α* Expression and Stabilization

First, we investigated the impact of oxygen and compressive force treatment on gene expression of *Hif-1α*. Neither reduced oxygen supply (*p* = 0.0578) alone nor compressive force treatment under normoxic (*p* = 0.2190) or hypoxic conditions (*p* = 0.8993) affected *Hif-1α* gene expression (Figure 2(a)). Accordingly, we detected no changes in *Hif-1α* gene expression with pressure on gas-impermeable plates (*p* = 0.9872) or gas-permeable plates (*p* = 0.7417, Figure 2(b)). HIF-1*α* protein was stabilized under normoxic conditions with pressure application (*p* = 0.0014, Figure 2(c)). As expected, more HIF-1*α* protein was detectable under hypoxic conditions (*p* < 0.0001). Surprisingly, this stabilization of HIF-1*α* was prevented, when compressive force was applied under hypoxic conditions in macrophages (*p* < 0.0001; Figure 2(c)). In contrast to gas-impermeable plates, where we could detect HIF-1*α* stabilization (*p* = 0.0251), usage of gas-permeable plates significantly prevented HIF-1*α* protein stabilization after pressure application (*p* = 0.0012; Figure 2(d)).

### 3.2. Effects of Mechanical Strain (Pressure) and Oxygen Supply on Gene Expression of Inflammatory Genes

Next, we investigated the gene expression of inflammatory genes *tumor necrosis factor* (*Tnf*) and *interleukin-6* (*Il-6*), which are known to play an important role during orthodontic tooth movement, but are not common HIF-1*α* target genes.

*Tnf* gene expression was enhanced after compressive force treatment under normoxic conditions (*p* = 0.0002). Hypoxia reduced *Tnf* gene expression under control conditions (*p* = 0.0022) and with pressure treatment (*p* = 0.0002; Figure 3(a)). Of note, an inductive effect of compressive force was still detectable under hypoxic conditions (*p* = 0.0030; Figure 3(a)). Accordingly, we observed enhanced *Tnf* gene expression on gas-impermeable plates (*p* < 0.0001; Figure 3(b)). This effect was not detectable with gas-permeable plates (*p* = 0.7721) leading to a significant effect of oxygen supply under compressive force treatment (*p* < 0.0001; Figure 3(b)).

Gene expression of *Il-6* was significantly elevated after pressure application in macrophages under normoxic conditions (*p* = 0.0005, Figure 3(c)) and on gas-impermeable plates (*p* < 0.0001, Figure 3(d)). Hypoxia increased *Il-6* gene expression without pressure application (*p* = 0.0046), while the pressure-derived effect was no longer observed (*p* = 0.9983; Figure 3(c)). Reduction of oxygen thereby seems to prevent the mechanotransductive effect on *Il-6* expression (*p* = 0.0097; Figure 3(c)). Improved oxygen supply due to gas-permeable plates restored the pressure effect on *Il-6* gene expression (*p* = 0.0374; Figure 3(d)). Nevertheless, we observed a significantly reduced *Il-6* expression after compressive force treatment on gas-permeable plates (*p* = 0.0099; Figure 3(d)).

### 3.3. Effects of Mechanical Strain (Pressure) and Oxygen Supply on the Expression of HIF-1*α* Target Genes Involved in Orthodontic Tooth Movement

*Matrixmetalloproteinase-9* (*Mmp-9*) is involved in extracellular matrix reorganization, which takes place during orthodontic tooth movement, and its expression is known to be controlled by HIF-1*α*. Therefore, we tested if *Mmp-9* expression is affected by pressure or oxygen supply. *Mmp-9* gene expression was elevated after compressive force application under normoxic conditions (*p* = 0.0058; Figure 4(a)) and on gas-impermeable plates (*p* = 0.0068; Figure 4(b)). As expected, hypoxia increased *Mmp-9* gene expression without additional compressive force treatment (*p* = 0.0137), while no additional effect of pressure was detectable under hypoxic conditions (*p* = 0.7796; Figure 4(a)). On gas-permeable plates, compressive force enhanced *Mmp-9* gene expression (*p* = 0.0008), while improved oxygen supply did not impact on *Mmp-9* expression under control (*p* = 0.9849) or pressure conditions (*p* = 0.3005; Figure 4(b)).

Next, we investigated gene expression of *cyclooxygenase-2* (*Cox-2*), which is regulated by HIF-1*α* under hypoxia and involved in orthodontic tooth movement by enhancing production of prostaglandin E2 (PG-E2). *Cox-2* expression was increased after pressure application under normoxic (*p* = 0.0197) and hypoxic conditions (*p* = 0.0017; Figure 5(a)). As expected, *Cox-2* expression was elevated under hypoxic conditions without (*p* = 0.0235) and with additional mechanical strain (*p* = 0.0035; Figure 5(a)). According to the gene expression data, we detected enhanced PG-E2 secretion after compressive force treatment under normoxic (*p* = 0.0003) and hypoxic conditions (*p* < 0.0001; Figure 5(b)). Hypoxia elevated PG-E2 secretion without (*p* < 0.0001) and with additional compressive force treatment (*p* = 0.0003; Figure 5(b)). Pressure application increased *Cox-2* gene expression even on gas-permeable plates with improved oxygen supply (*p* = 0.0067; Figure 5(c)). In line with that, improved oxygen supply during compressive force treatment did not impact on increased PG-E2 secretion (*p* = 0.0924; Figure 5(d)).

One main purpose of HIF-1*α* is to improve oxygen supply during hypoxic conditions. Therefore, the expression of vascular endothelial growth factor (VEGF), which is responsible for angiogenesis, is controlled among others by HIF-1*α*. Under normoxic conditions, pressure application was associated with enhanced *Vegf* gene expression (*p* = 0.0024). Hypoxia increased *Vegf* under control conditions without compressive force application (*p* = 0.0052; Figure 6(a)). Under hypoxic conditions, pressure application failed to induce *Vegf* gene expression (*p* = 0.6450; Figure 6(a)).

Accordingly, VEGF protein secretion was increased after pressure application under normoxic (*p* > 0.0001) and hypoxic conditions (*p* > 0.0001; Figure 6(b)). Hypoxia itself increased VEGF secretion without (*p* = 0.0002) and with compressive force treatment (*p* > 0.0001; Figure 6(b)). We detected elevated *Vegf* gene expression on gas-impermeable plates after pressure treatment (*p* < 0.0001). This effect was circumvented on gas-permeable plates (*p* < 0.0001; Figure 6(c)). Improved oxygen supply showed no effect on VEGF secretion, as we detected an inductive pressure effect on gas-impermeable (*p* > 0.0001) and gas-permeable plates (*p* > 0.0001; Figure 6(d)).

## 4. Discussion

The aim of this study was to examine the effects of oxygen supply versus mechanical strain (pressure) occurring during orthodontic tooth movement (OTM) on macrophages. Therefore, we investigated stabilization of HIF-1*α* in macrophages and expression of genes involved in OTM and of common HIF-1*α* target genes and proteins.

Normally, HIF-1*α* is stabilized under hypoxia to counteract these hypoxic conditions and to improve cell survival [2, 24]. Due to the constant expression of the *Hif-1α* mRNA, HIF-1*α* protein is continuously formed [12]. Depending on the surrounding oxygen content, it is degraded under normoxic conditions and stabilized under hypoxia. HIF-1*α* degradation is initiated by an oxygen-dependent hydroxylation of its proline and asparagine amino acids, whereupon the Von Hippel Lindau protein can bind to HIF-1*α*. Subsequent ubiquitination then enables degradation of HIF-1*α* in the proteasome [12]. In line with that, we found no changes in *Hif-1α* gene expression under hypoxic conditions or during pressure application, although we detected more HIF-1*α* protein upon both tested conditions with a combination of hypoxia and compressive force treatment preventing HIF-1*α* stabilization. Next to oxygen content, HIF-1*α* degradation was reported to be controlled by other mechanisms [25, 26]. These authors found that with the withdrawal of glucose, less HIF-1*α* was stabilized than in an ischemia control group, despite ischemia. The authors also hypothesize that the transcriptional activity of HIF-1*α* is less induced by severe ischemia than by a slight lack of oxygen [25]. Furthermore, it was reported that enhanced Na^+^ levels under inflammatory conditions were accompanied by HIF-1*α* protein stabilization [26]. The usage of gas-permeable plates prevented the stabilizing effect of compressive force treatment, indicating that stabilization of HIF-1*α* protein after pressure application in macrophages was rather triggered by the reduced oxygen supply than via mechanotransduction by the mechanical strain (pressure) applied. These results were contrary to data obtained with periodontal ligament fibroblasts [17]. Using a similar setup with gas-permeable plates, these were found to stabilize HIF-1*α* protein mainly via mechanotransduction due to the mechanical strain (pressure) applied with oxygen supply playing only a minor role on HIF-1*α* stabilization [17].

*Tumor necrosis factor* (*Tnf*) and *interleukin 6* (*Il-6*) belong to the group of proinflammatory cytokines, which are, among others, produced by macrophages [27]. The inflammatory response that occurs during orthodontic tooth movement (OTM) usually takes place under sterile conditions [1]. Damage-associated molecular patterns are released by necrotic cell death so that the inflammatory cascade can begin [28]. As an initial reaction, cytokines such as TNF and IL-6 are secreted [7]. Both inflammation markers are significantly involved in OTM but are not known classical HIF-1*α* target genes [29]. Both HIF-1*α* and TNF were increased in gingival crevicular fluid both in chronic and in aggressive forms of periodontitis [30]. In this study, pressure application increased *Tnf* and *Il-6* gene expression, while reduction of oxygen reduced gene expression of both genes in macrophages. Of note, compressive force still enhanced *Tnf*, but not *Il-6* mRNA even under hypoxic conditions. Surprisingly, improved oxygen supply during mechanical strain prevented an upregulation of *Tnf* gene expression, while *Il-6* gene expression was still elevated, indicating a regulatory role of oxygen supply or HIF-1*α* stabilization in the regulation of *Tnf*. It is already known that TNF stabilizes HIF-1*α* in tubular cells [31]. Judging from the results, proinflammatory cytokine secretion would be mainly due to mechanical compression, which could be partially compensated by improved oxygenation. Periodontal ligament fibroblasts and dental pulp cells were reported to react to mechanical compression by upregulating IL-6 [29, 32] documenting an important role in tooth movement and bone remodelling.

Matrix metalloproteinases (MMPs) are also involved in these remodelling processes and are mainly responsible for the breakdown of the extracellular matrix during orthodontic tooth movement [1, 33, 34]. Macrophages react to pressure application and hypoxia with an upregulation of *Mmp-9* gene expression, which is known to be regulated by HIF-1*α* [35]. As *Mmp-9* remained increased even with improved oxygen supply upon compressive force treatment, it seemed unlikely that upregulation is mediated via HIF-1*α* in the context of pressure application. Next to HIF-1*α*, *Mmp-9* expression could also be regulated by reactive oxygen species and TNF [36].

Another HIF-1*α* target gene is *cyclooxygenase-2* (*Cox-2*), which in contrast to the constitutively expressed COX-1 is produced during inflammation and catalyses the formation of prostaglandins from arachidonic acid [27]. Prostaglandin-E2 (PG-E2) can increase vascular permeability on site and has a chemotactic effect. It can also promote bone resorption by promoting the formation of osteoclasts via the RANKL/OPG cascade [17, 27]. *Cox-2* gene expression was rapidly enhanced after 2 h of pressure application [7]. Here, we detected an increase of *Cox-2* and PG-E2 with compressive force, which was even potentiated by hypoxic conditions. An improved oxygen supply nevertheless showed pressure induction in both gene and protein expression, although HIF-1*α* was not stabilized. Next to HIF-1*α*, *Cox-2* gene expression can also be controlled by NF-*κ*B [32]. In line with the data obtained in macrophages, *Cox-2* gene expression was not affected by improved oxygen supply during compressive force treatment [17].

The main purpose of HIF-1*α* is to improve oxygen supply in tissues [24]. Therefore, HIF-1*α* controls expression of vascular endothelial growth factor (VEGF), which is described in the literature as the most important mediator of angiogenesis and vascular permeability [2, 12, 14, 24]. In this study, VEGF gene and protein expression was increased by pressure induction as well as by hypoxia, with a potentiating effect, when combining both factors. These results were also associated with HIF-1*α* stabilization. In spite of an improved oxygen supply, a significant pressure effect on the protein level could be shown, which was, however, less pronounced than on gas-impermeable plates.

## 5. Conclusions

With our study, we were able to further elucidate the complex multicellular pseudoinflammatory processes and their regulation within the periodontal ligament enabling therapeutic orthodontic tooth movement (OTM). Mechanical compressive strain as occurring during OTM in pressure zones of the periodontal ligament has a mechanotransductive effect on compressed cells and reduces oxygen supply by compression of blood vessels. In this study, we separated and uncoupled both effects by using gas-permeable compared to gas-impermeable plates and by inducing hypoxia compared to normoxia. HIF-1*α* was not stabilized by compressive force treatment on gas-permeable plates, which provide sufficient oxygen supply. This indicates that in macrophages HIF-1*α* stabilization is rather induced via the decreased oxygen supply associated with OTM than via mechanotransduction by mechanical strain. As the opposite is true in periodontal ligament fibroblasts, as reported before, our results thus indicate that both cell types involved in the mediation of orthodontic tooth movement are affected and respond differently to hypoxic conditions and mechanical compressive strain, which occur concomitantly during OTM, thus indicating different roles in the regulation of OTM at the cellular-molecular level.

## Figures and Tables

**Table 1:** RT-qPCR gene and primer specifications for reference (*Eef*1*a*1, *Sdha*) and target genes.

Gene symbol	Gene name	Accession number	5′-forward primer-3′	5′-reverse primer-3′
*Eef1a1*	Eukaryotic translation elongation factor 1 alpha 1	NM_010106.2	AAAACATGATTACAGGCACATCCC	GCCCGTTCTTGGAGATACCAG
*Sdha*	Succinate dehydrogenase complex, subunit A	NM_023281.1	AACACTGGAGGAAGCACACC	AGTAGGAGCGGATAGCAGGAG
*Cox-2*	Cyclooxygenase-2	NM_011198.4	TCCCTGAAGCCGTACACATC	TCCCCAAAGATAGCATCTGGAC
*Hif-1α*	Hypoxia inducible factor 1*α*	NM_001313919.1	CCAAGGAGCCTTAACCTGTCTG	CGCTTCCTCTGAGCATTCTGC
*Il-6*	Interleukin-6	NM_031168.2	ACAAAGCCAGAGTCCTTCAGAG	GAGCATTGGAAATTGGGGTAGG
*Mmp-9*	Matrix metalloproteinase 9	NM_013599.4	GTGGGGTTTCTGTCCAGACC	GCACGCTGGAATGATCTAAGC
*Tnf*	Tumor necrosis factor	NM_013693.3	TCGAGTGACAAGCCTGTAGCC	CTTTGAGATCCATGCCGTTGGC
*Vegf*	Vascular endothelial growth factor	NM_001287056.1	ACAAGCCTGTAGCCCACGTC	TTGGTTGTCTTTGAGATCCCATGCC

## Data Availability

All datasets are publically available upon request from the corresponding author.

## Supplementary Materials

Supplementary Materials Material and methods in vitro cell culture experiments.
