# Supplementary material for: Role of Oxygen Supply in Macrophages in a Model of Simulated Orthodontic Tooth Movement
Source: Mediators Inflamm. 2020 Jul 29;2020:5802435. doi: 10.1155/2020/5802435 (PMC7424081; doi:10.1155/2020/5802435)
Supplement: Supplementary Materials — Material and methods in vitro cell culture experiments. [file 5802435.f1.pdf]

Supplemental information

Material and Methods

In vitro cell culture experiments

Experiment 3: Effects of mechanical strain (pressure) with DMOG treatment on macrophages
DMOG (Dimethyloxaloylglycine) is an inhibitor for prolyl hydroxylase leading to HIF-1α protein stabilisation. To uncouple reduced oxygen supply and effects of enhanced HIF-1α protein we incubated 250,000/ml RAW264.7 macrophages after 24 h of preincubation without and with 400 µM DMOG without and with pressure application for 4 h (Supplemental figures 2-7) and repeated Experiment 2 with corresponding compressive force treatment for 4h (Supplemental figures 8-11).

Supplemental figures

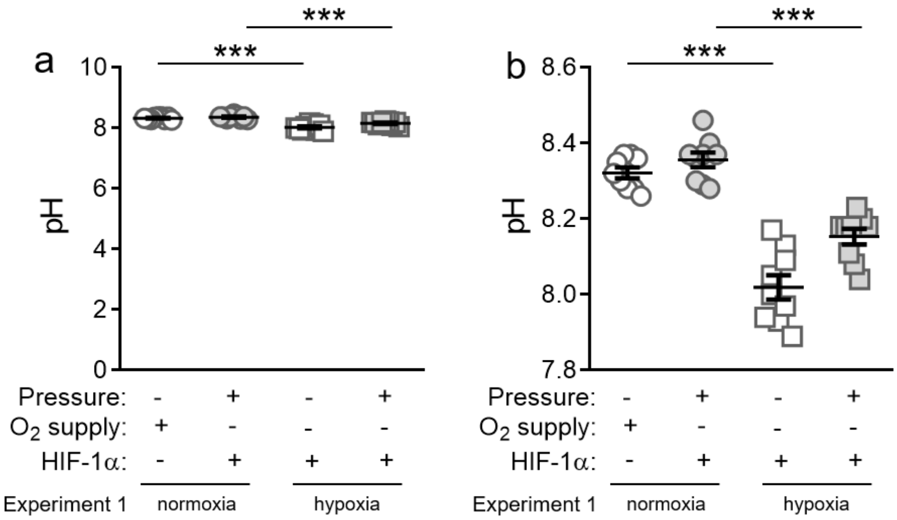

Supplemental figure 1: Measurement of pH in the cell culture supernatant scaled across the entire pH scale (a) and the relevant pH range magnified (b). Of note, measured pH was over 8, while DMEM should be around 7.0-7.6 according to the manufacturer's datasheet. This might be due to the supplementation of FCS (pH up to 8.5), but as we measured all pH values of the various setups and experiments in one batch, pH differences between groups should be representative, thus showing that the carbon dioxide and associated pH changes do not seem to play a major role in the issue investigated. *Statistics:* ANOVA followed by Holm Sidak´s multiple comparison tests; AU = arbitrary units; ***p $\leq$ 0.001.

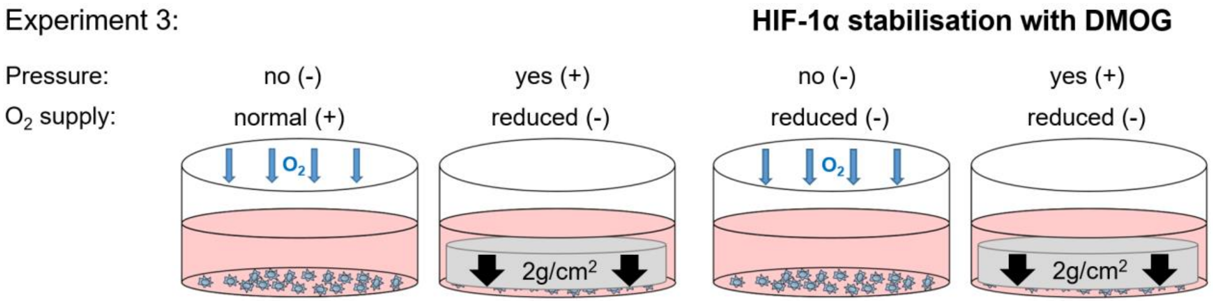

Supplemental figure 2: Schematic representation of experimental settings affecting HIF-1α stabilistation and mechanical strain (pressure) on adherently growing macrophages.

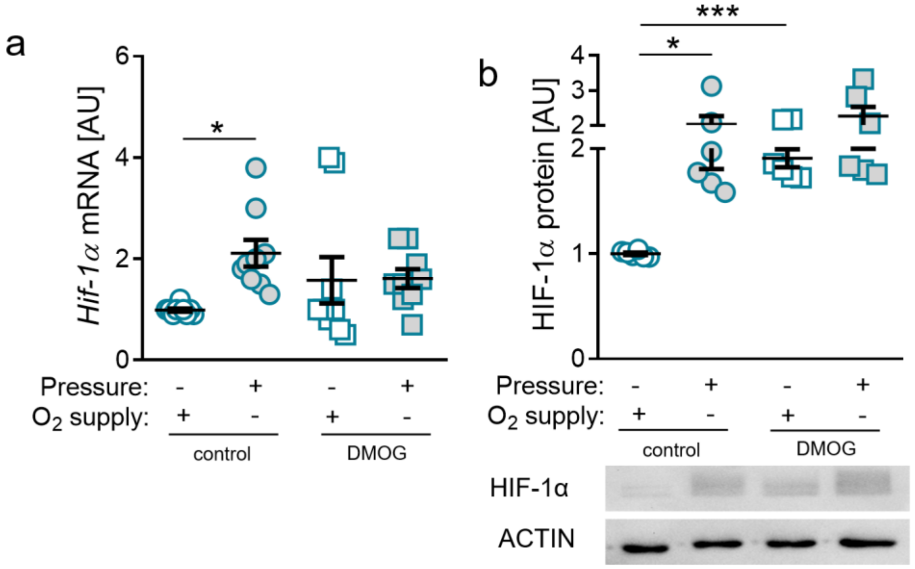

Supplemental figure 3: *Hif-1α* gene (a) and protein (b) expression without and with DMOG treatment without or in combination with compressive force treatment. *Below:* representative immunoblot. (*Statistics:* Welch-corrected ANOVAs followed by Tamhane´s T2 multiple comparison; AU = arbitrary units; *$p \leq 0.05$, ***$p \leq 0.001$.

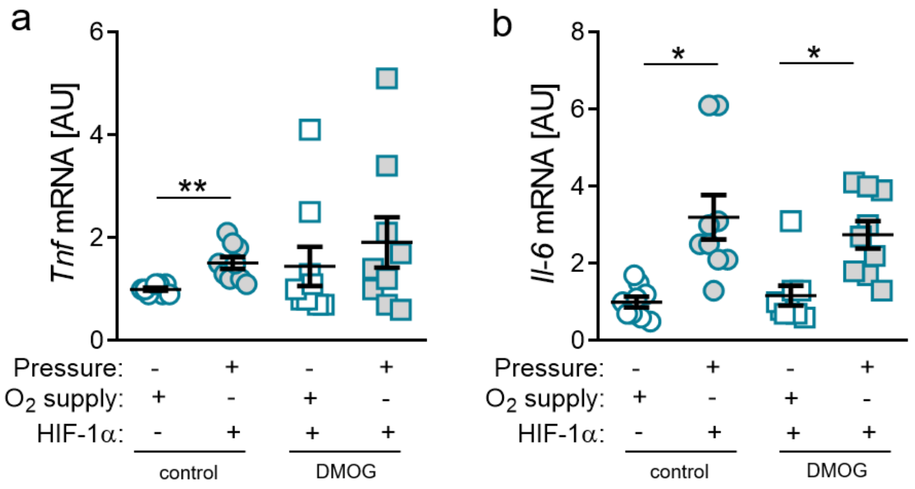

Supplemental figure 4: *Tnf* (a) and *Il-6* (b) gene expression without and with DMOG treatment without or in combination with compressive force treatment. *Statistics:* Welch-corrected ANOVAs followed by Tamhane´s T2 multiple comparison; AU = arbitrary units; *$p \leq 0.05$; **$p \leq 0.01$.

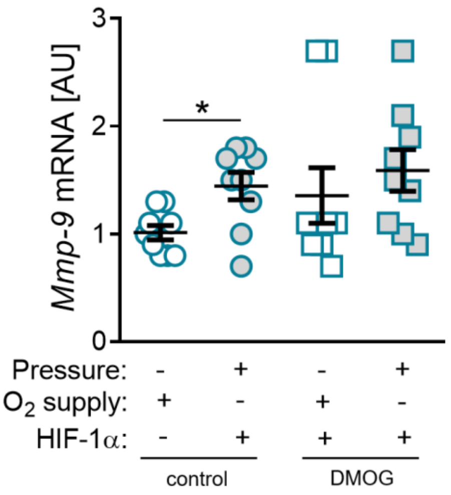

Supplemental figure 5: (a) *Mmp-9* gene expression without and with DMOG treatment without or in combination with compressive force treatment. *Statistics:* Welch-corrected ANOVAs followed by Tamhane´s T2 multiple comparison; AU = arbitrary units; *$p \leq 0.05$; **$p \leq 0.01$; ***$p \leq 0.001$.

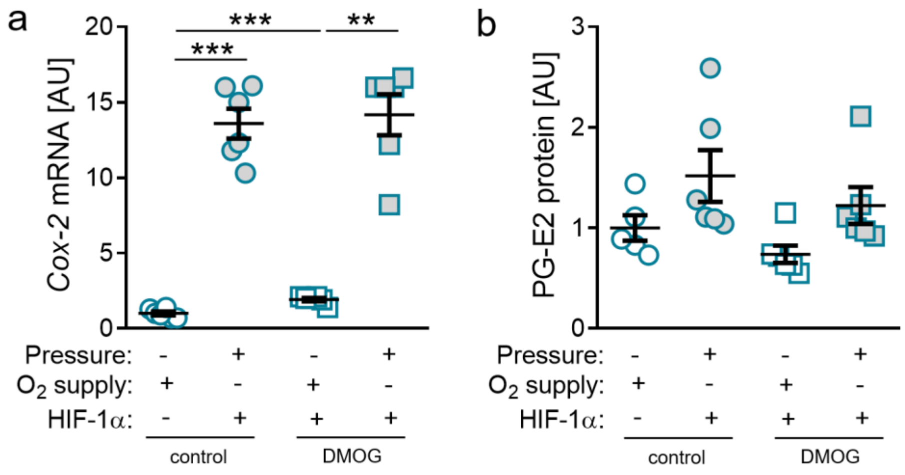

Supplemental figure 6: *Cox-2* gene expression and PG-E2 secretion without and with DMOG treatment without or in combination with compressive force treatment. *Statistics:* Welch-corrected ANOVAs followed by Tamhane´s T2 multiple comparison; AU = arbitrary units; **p $\leq$ 0.01; ***p $\leq$ 0.001.

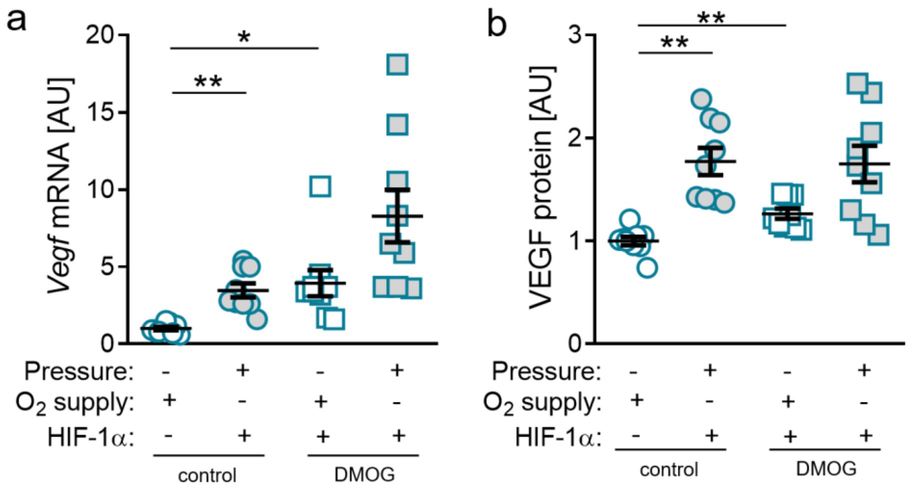

Supplemental figure 7: *Vegf* gene expression (a) and VEGF secretion (b) without and with DMOG treatment without or in combination with compressive force treatment. *Statistics:* ANOVA followed by Holm Sidak´s or Tamhane´s T2 multiple comparison tests; AU = arbitrary units; **p $\leq$ 0.01; ***p $\leq$ 0.001.

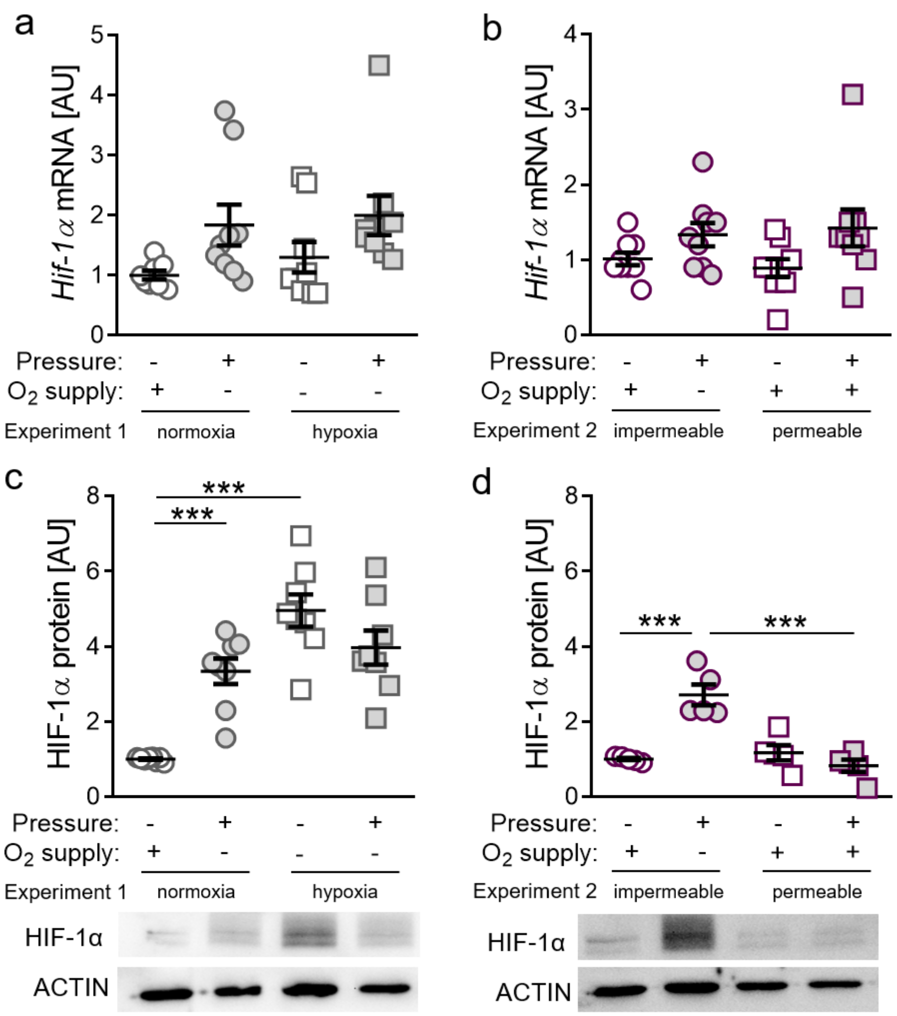

Supplemental figure 8: (a) *Hif-1α* gene expression under normoxic or hypoxic cell culture conditions with or without compressive force treatment for 4 h. (b) *Hif-1α* gene expression on gas-impermeable or gas-permeable plates with or without pressure application for 4 h. (c) HIF-1α protein expression under normoxic or hypoxic cell culture conditions with or without compressive force treatment for 4 h. *Below:* representative immunoblot. (d) HIF-1α protein expression on gas-impermeable or gas-permeable plates with or without pressure application. *Below:* representative immunoblot. *Statistics:* ANOVA followed by Holm Sidak´s or Tamhane´s T2 multiple comparison tests; AU = arbitrary units; ***$p \leq 0.001$.

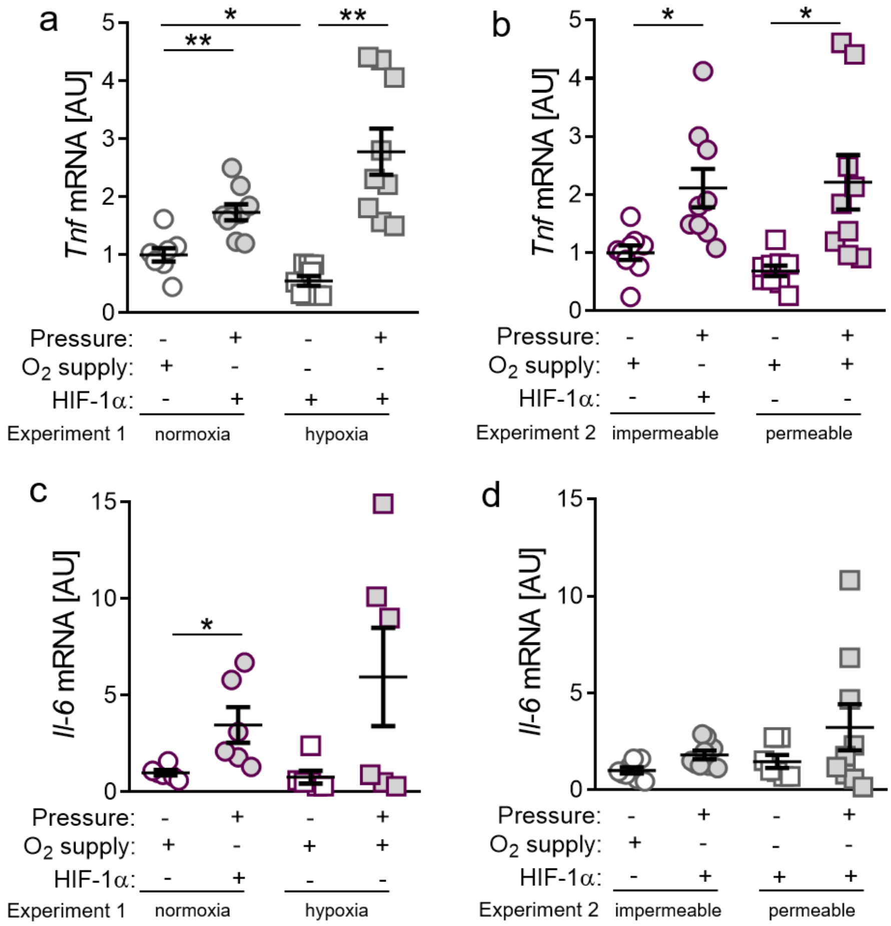

Supplemental figure 9: (a) *Tnf* gene expression under normoxic or hypoxic cell culture conditions with or without compressive force treatment for 4 h.. (b) *Tnf* gene expression on gas-impermeable or gas-permeable plates with or without pressure application for 4 h.. (c) *Il-6* gene expression under normoxic or hypoxic cell culture conditions with or without compressive force treatment for 4 h. (d) *Il-6* gene expression on gas-impermeable or gas-permeable plates with or without pressure application for 4 h. *Statistics:* Welch-corrected ANOVAs followed by Tamhane´s T2 multiple comparison; AU = arbitrary units; *p $\leq$ 0.05; **p $\leq$ 0.01.

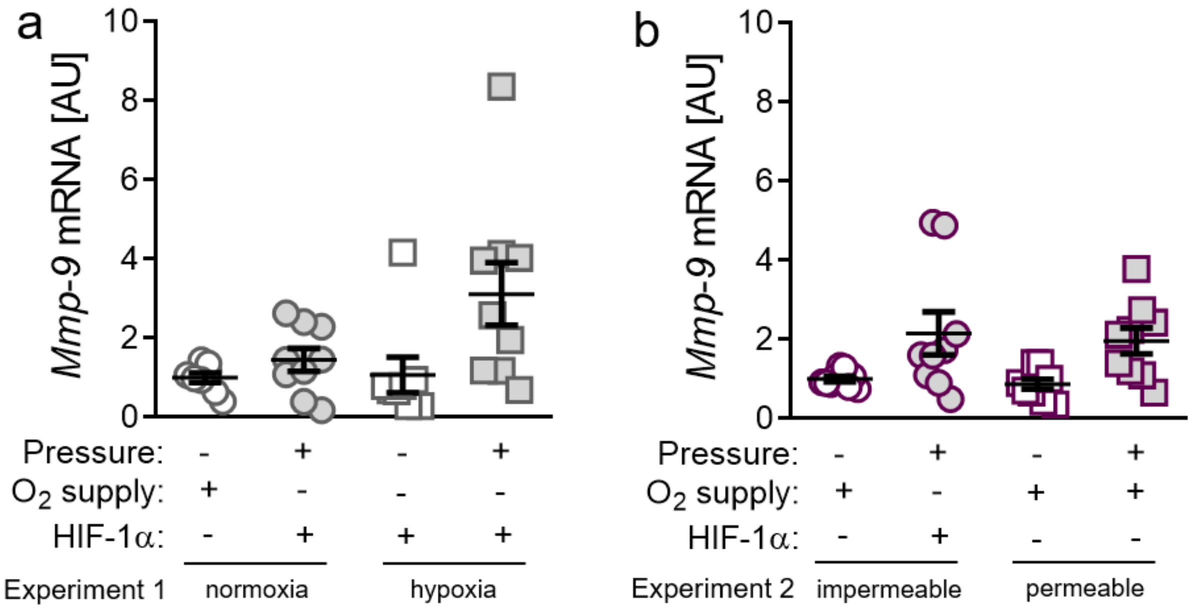

Supplemental figure 10: (a) *Mmp-9* gene expression under normoxic or hypoxic cell culture conditions with or without compressive force treatment for 4 h. (b) *Mmp-9* gene expression on gas-impermeable or gas-permeable plates with or without pressure application for 4 h. *Statistics:* Welch-corrected ANOVAs followed by Tamhane´s T2 multiple comparison; AU = arbitrary units.

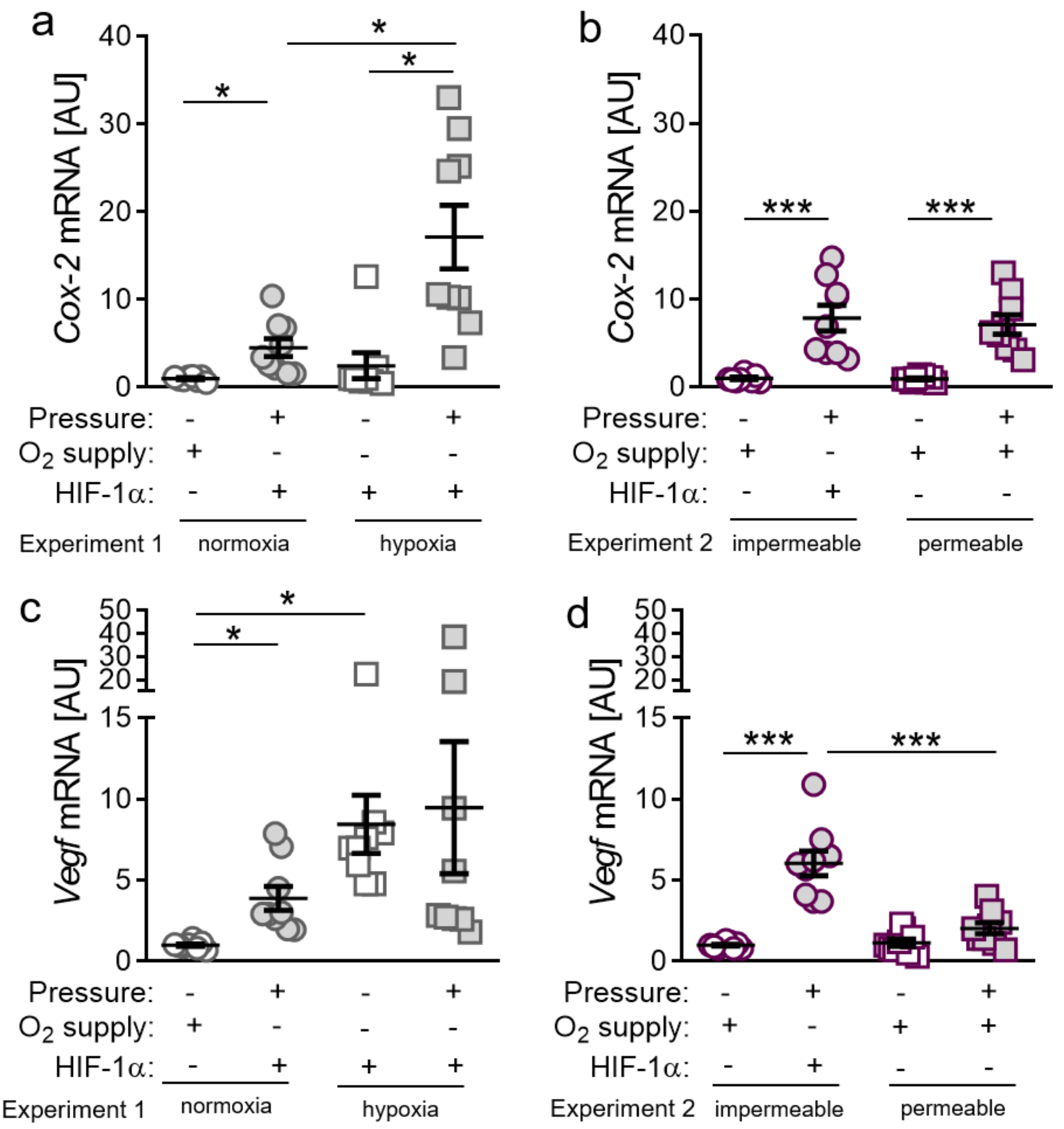

Supplemental figure 11: (a) *Cox-2* gene expression under normoxic or hypoxic cell culture conditions with or without compressive force treatment for 4 h. (b) *Cox-2* gene expression on gas-impermeable or gas-permeable plates with or without pressure application for 4 h. (c) *Vegf* gene expression under normoxic or hypoxic cell culture conditions with or without compressive force treatment for 4 h. (d) *Vegf* gene expression on gas impermeable or gas permeable plates with or without pressure application for 4 h. *Statistics:* ANOVA followed by Holm Sidak´s or Tamhane´s T2 multiple comparison tests; AU = arbitrary units; *$p \leq 0.05$; ***$p \leq 0.001$.
